# Sodium Niobate with a Large Interlayer Spacing: A Fast‐Charging, Long‐Life, and Low‐Temperature Friendly Lithium‐Storage Material

**DOI:** 10.1002/advs.202300583

**Published:** 2023-04-29

**Authors:** Jiazhe Gao, Liting Yang, Cihui Huang, Guisheng Liang, Yi Lei, Songjie Li, Wenze Wang, Yinjun Ou, Shangfu Gao, Xuehua Liu, Yifeng Cheng, Jincang Zhang, Zhongzhu Liu, Aiming Guo, Robson Monteiro, Luanna Parreira, Rogerio Ribas, Chunfu Lin, Limin Wu, Renchao Che

**Affiliations:** ^1^ Laboratory of Advanced Materials, Shanghai Key Lab of Molecular Catalysis and Innovative Materials, Academy for Engineering & Technology Fudan University Shanghai 200438 China; ^2^ Institute of Materials for Energy and Environment, School of Materials Science and Engineering Qingdao University Qingdao 266071 China; ^3^ School of Materials Science and Engineering Hainan University Haikou 570228 China; ^4^ Zhejiang Laboratory Hangzhou 311100 China; ^5^ CITIC Metal Co.Ltd. Beijing 122099 China; ^6^ Companhia Brasileira de Metalurgia e Mineração (CBMM) Gerais 38183903 Brazil; ^7^ Inner Mongolia University Hohhot 010021 China

**Keywords:** in situ characterization, interlayer spacing, lithium‐storage mechanism, niobate, temperature‐dependent lithium‐storage property

## Abstract

Niobate Li^+^‐storage anode materials with shear ReO_3_ crystal structures have attracted intensive attention due to their inherent safety and large capacities. However, they generally suffer from limited rate performance, cyclic stability, and temperature adaptability, which are rooted in their insufficient interlayer spacings. Here, sodium niobate (NaNb_13_O_33_) micron‐sized particles are developed as a new anode material owning the largest interlayer spacing among the known shear ReO_3_‐type niobates. The large interlayer spacing of NaNb_13_O_33_ enables very fast Li^+^ diffusivity, remarkably contributing to its superior rate performance with a 2500 to 125 mA g^−1^ capacity percentage of 63.2%. Moreover, its large interlayer spacing increases the volume‐accommodation capability during lithiation, allowing small unit‐cell‐volume variations (maximum 6.02%), which leads to its outstanding cyclic stability with 87.9% capacity retention after as long as 5000 cycles at 2500 mA g^−1^. Its cyclic stability is the best in the research field of niobate micron‐sized particles, and comparable to that of “zero‐strain” Li_4_Ti_5_O_12_. At a low temperature of −10 °C, it also exhibits high rate performance with a 1250 to 125 mA g^−1^ capacity percentage of 65.6%, and even better cyclic stability with 105.4% capacity retention after 5000 cycles at 1250 mA g^−1^. These comprehensively good electrochemical results pave the way for the practical application of NaNb_13_O_33_ in high‐performance Li^+^ storage.

## Graphical abstract

NaNb_13_O_33_ micron‐sized particles are explored as a practical Li^+^‐storage anode material with comprehensively good electrochemical properties and broad temperature adaptability. This new niobate owns the largest interlayer spacing among the known shear ReO_3_‐type niobates. This merit leads to very fast Li^+^ diffusivity, notable intercalation‐pseudocapacitive behavior, and small unit‐cell‐volume variations, greatly benefiting the rate performance, cyclic stability, and low‐temperature electrochemical properties.

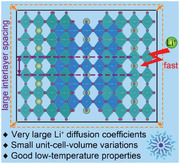

## 1 Introduction

Lithium‐ion batteries (LIBs) have been widely used for various devices and equipment, such as battery electric vehicles (BEVs),^[^ ^1^ ^]^ which require higher‐performance electrode materials.^[^ ^2^ ^]^ The traditional and cheap graphite anode material shows a large theoretical capacity of 372 mAh g^−1^ and practical capacity of 300–360 mAh g^−1^.^[^ ^3^ ^]^ However, its wide applications are limited by its unsatisfactory rate and safety performance.^[^ ^4^ ^]^ The popular Li_4_Ti_5_O_12_ with a plateau at 1.55 V (vs Li/Li^+^) serves as a safe anode material, but suffers from its small capacity (theoretically 175 mAh g^−1^ and practically ≈170 mAh g^−1^).^[^ ^5^ ^]^ In addition, both materials deliver poor low‐temperature electrochemical properties, limiting their applications in high latitudes, high altitudes, and winter periods.^[^ ^6^ ^]^ Thus, it becomes highly desirable to explore new anode materials with comprehensively good electrochemical properties and broad temperature adaptability.

Recently, niobates have been regarded as promising anode materials with high performance.^[^ ^7^ ^]^ Nb is not a rare metal, and its amount in the earth's crust is comparable to that of Li and Pb.^[^ ^8^ ^]^ The active Nb^4+^/Nb^5+^ and Nb^3+^/Nb^4+^ redox couples enable not only safe operating potentials but also large theoretical capacities. Moreover, the large anion versus cation ratios in niobates lead to open crystal structures, such as shear ReO_3_ crystal structures. The structural units of the shear ReO_3_ structures are generally constructed by corner‐sharing octahedron‐blocks, which are connected to neighboring blocks via octahedron edge‐sharing.^[^ ^9^ ^]^ Such edge‐sharing can stabilize the formed A–B–A interlayer structure during Li^+^ insertion. However, the interlayer spacings of previously‐reported shear ReO_3_‐type niobates are insufficient. For instance, the popular TiNb_2_O_7_ has an interlayer spacing of only 3.7989 Å, which limits its Li^+^ transport and thus rate performance.^[^ ^10^ ^]^ In addition, the inferior volume‐buffering capability rooted in its smaller interlayer spacing leads to relatively large unit‐cell‐volume expansion (7.22%) after lithiation to 1.0 V and thus insufficient cyclic stability.^[^ ^11^ ^]^ These issues hinder its Li^+^‐storage applications, especially when its electrode loading is large. The popular nanostructure construction is an extrinsic solution that can tackle the above two issues, because nanomaterials have not only small primary particles with small Li^+^ transport lengths but also abundant nanopores for accommodating primary‐particle expansion.^[^ ^12^ ^]^ Unfortunately, nanomaterials are generally of high production cost and low tap densities. Clearly, a better and intrinsic solution is to explore new niobates with large interlayer spacings.

Here, we design and explore sodium niobate (NaNb_13_O_33_, theoretical capacity: 396 mAh g^−1^ based on Nb^5+^↔Nb^3+^) as a new niobate for Li^+^ storage, which owns the largest interlayer spacing (3.8484 Å) among those of previously‐reported shear ReO_3_‐type niobates. Thus, the Li^+^ diffusivity can be enhanced and the maximum unit‐cell‐volume variation can be decreased,^[^ ^13^ ^]^ benefiting not only the rate performance and cyclic stability but also the low‐temperature electrochemical properties. In this work, we successfully prepare pure NaNb_13_O_33_ micron‐sized particles through a two‐step solid‐state reaction method. The Li^+^‐storage properties and mechanisms of NaNb_13_O_33_ at room and low temperatures are intensively studied. At 25 °C, NaNb_13_O_33_ delivers very fast Li^+^ diffusivity, remarkably contributing to its outstanding rate performance with a 1250 mA g^−1^/2500 mA g^−1^ to 125 mA g^−1^ capacity percentage of 71.8%/63.2%. Very excitingly, NaNb_13_O_33_ exhibits superior cyclic stability with 87.9% capacity retention after as long as 5000 cycles at 2500 mA g^−1^ owing to its maximum unit‐cell‐volume variation of only 6.02% at 0.8 V. At −10 °C, NaNb_13_O_33_ also delivers high rate performance with a 1250 to 125 mA g^−1^ capacity percentage of 65.6%, and superior cyclic stability with 105.4% capacity retention after 5000 cycles at 1250 mA g^−1^. Hence, NaNb_13_O_33_ can be an ideal anode material for LIBs of BEVs, even when its particles are micron‐sized.

## 2 Results and Discussion

### 2.1 Physico‐Chemical Characterizations

**Figure 1:**
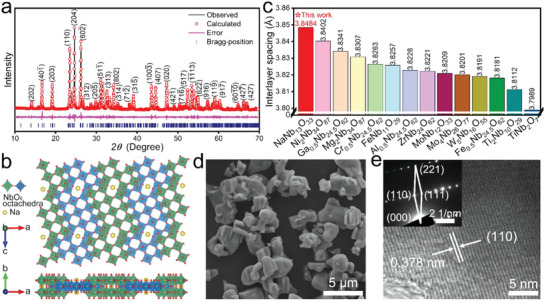
Physico‐chemical characterizations of NaNb_13_O_33_. a) powder XRD pattern of NaNb_13_O_33_ with Rietveld refinement (main diffraction peaks are labeled). b) Crystal structure of NaNb_13_O_33_. c) Interlayer spacing of NaNb_13_O_33_, and its comparison with that of previously‐reported shear ReO_3_‐type niobates. d) FESEM image. e) HRTEM image (inset: SAED pattern).

The Rietveld‐refined X‐ray diffraction (XRD) pattern (**Figure** **1**a) matches well with monoclinic NaNb_13_O_33_ (JCPDS 73–1788, space group of *C2/m*). The crystal structure of NaNb_13_O_33_ (Figure 1b) contains NbO_6_ octahedra spreading on different shear *ac*‐planes.^[^ ^14^ ^]^ The interlayers share both octahedron corners and edges. The interlayers are connected through sharing octahedron edges. Such octahedron arrangement guarantees an A–B–A interlayered structure with high stability. Interestingly, this special arrangement is regularly interrupted by Na^+^. The NaO_12_ decahedra serve as anchors connecting shear *ac*‐planes by edge‐sharing, which further enhances the structural stability. The refinement results (Tables S1 and S2, Supporting Information) reveal the lattice constants of *a* = 22.49614(68) Å, *b* = 3.84842(10) Å, *c* = 15.42272(42) Å, and *V* = 1334.800(83) Å^3^. Very excitingly, NaNb_13_O_33_ exhibits the largest *b* value among the known shear ReO_3_‐type niobate materials (Figure 1c; Table S3, Supporting Information), indicating its largest interlayer spacing (3.8484 Å), which is significantly larger than that of graphite (3.35 Å).^[^ ^15^ ^]^ As a result, very fast Li^+^ transport within the NaNb_13_O_33_ lattice can be achieved since it is known that even tiny lattice enlargement in intercalating electrode materials can obviously increase their Li^+^ diffusion coefficients.^[^ ^16^ ^]^

The NaNb_13_O_33_ sample shows micron‐sized particles (1–6 µm, Figure 1d) and a small specific surface area (0.444 m^2^ g^−1^ based on the Brunauer−Emmett−Teller (BET) model, Figure S1, Supporting Information). Interestingly, there are abundant nanopores exist within the particles (Figure S2, Supporting Information). The lattice‐fringe spacing of NaNb_13_O_33_ is determined to be 0.378 nm (Figure 1e), corresponding to its (110) plane. Its large interlayer spacing is directly revealed by its high‐angle annular dark‐field (HAADF)‐scanning transmission electron microscopy (STEM) image (Figure S3, Supporting Information). Its regular electron‐diffraction spots (Figure 1e inset) match with its (110), (111), and (221) planes, which verifies its monoclinic structure and space group of *C2/m*. The element distributions of Na, Nb, and O in NaNb_13_O_33_ very homogeneous (Figure S4, Supporting Information), confirming the high purity of NaNb_13_O_33_. The Na:Nb molar ratio in NaNb_13_O_33_ is determined to be 0.96:13 by the X‐ray fluorescence (XRF) test, which matches well with its theoretical ratio (1:13).

### 2.2 Temperature‐Dependent Li^+^‐Storage Properties

**Figure 2:**
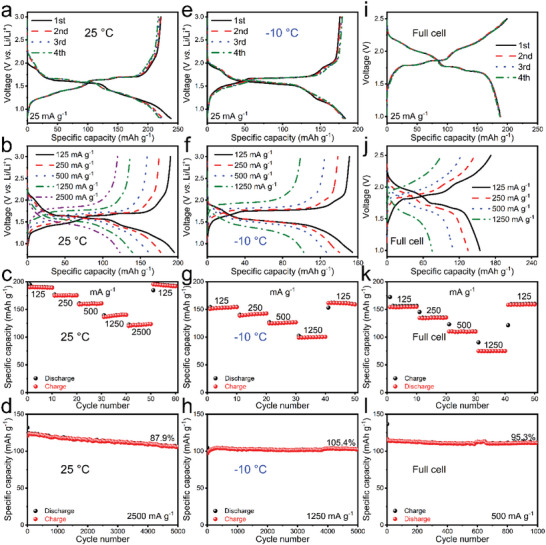
Temperature‐dependent electrochemical properties of NaNb_13_O_33_. a, e) First four‐cycle GCD curves of half cells at 25 mA g^−1^; b, f) GCD curves of half cells at various current rates; c, g) rate performance of half cells; d, h) long‐term cyclic stability of half cells after 5000 cycles at 25 and −10 °C. i) First four‐cycle GCD curves of NaNb_13_O_33_/LiFePO_4_ full cell at 25 mA g^−1^, j) GCD curves of NaNb_13_O_33_/LiFePO_4_ full cell at various current rates, k) rate performance of NaNb_13_O_33_/LiFePO_4_ full cell, l) long‐term cyclic stability of NaNb_13_O_33_/LiFePO_4_ full cell after 1000 cycles at 25 °C.

At 25 °C, the galvanostatic charge–discharge (GCD) curves of NaNb_13_O_33_ reveal a large first‐cycle discharge/charge capacity of 238.5/221.6 mAh g^−1^ with a high Coulombic efficiency of 92.9% at 25 mA g^−1^ (**Figure** **2**a). The average operating potential is ≈1.52 V during lithiation–delithiation, which is slightly lower than that of the popular Li_4_Ti_5_O_12_ (1.55 V).^[^ ^5^ ^]^ When increasing the current rate to 125, 250, 500, 1250, and 2500 mA g^−1^, NaNb_13_O_33_ is capable of retaining large reversible capacities of 190.1, 175.0, 159.0, 136.4, and 120.1 mAh g^−1^, respectively (Figure 2b,c), indicating its outstanding rate performance with a large 1250 mA g^−1^/2500 mA g^−1^ to 125 mA g^−1^ capacity percentage of 71.8%/63.2%. When turning the current rate back to 125 mA g^−1^, the capacity has no fading. NaNb_13_O_33_ exhibits good cyclic stability with 92.0% capacity retention after 200 cycles at 250 mA g^−1^ (Figure S5, Supporting Information). Ultra‐long‐term cycling tests are further performed at 2500 mA g^−1^, showing 87.9% retention after 5000 cycles (Figure 2d), which is the best result obtained from the known shear ReO_3_‐type niobates (Table S4, Supporting Information).

At a low temperature of −10 °C, the average operating potential of NaNb_13_O_33_ slightly increases to 1.59 V (Figure 2e). Its reversible capacity at 25 mA g^−1^ is 176.1 mAh g^−1^ (Figure 2e), retaining up to 79.5% of that at 25 °C. The first‐cycle Coulombic efficiency obviously increases to 96.1% (Figure 2e), which is originated from the formation of thinner solid‐electrolyte interphase (SEI) films at −10 °C (Figure S6, Supporting Information).^[^ ^17^ ^]^ At 125, 250, 500, and 1250 mA g^−1^, 151.0, 138.7, 124.8, and 99.0 mAh g^−1^ remain, respectively (Figure 2f,g). The 1250 to 125 mA g^−1^ capacity percentage at −10 °C reaches 65.6%, only slightly smaller than that at 25 °C (71.8%). This rate performance is remarkably higher than that of graphite with 5C to 0.5C capacity percentages of only 27.8% at −10 °C,^[^ ^13^ ^]^ and that of Li_4_Ti_5_O_12_ nano‐sized particles with no capacity at 5C and −10 °C.^[^ ^18^ ^]^ Moreover, NaNb_13_O_33_ still delivers excellent cyclic stability at −10 °C without any capacity decay (105.4% capacity retention) after 5000 cycles at 1250 mA g^−1^ (Figure 2h). This slight capacity increase could be rooted in the gradual NaNb_13_O_33_ activation because the full electrolyte soaking needs relatively long time at the low temperature.^[^ ^19^ ^]^

To demonstrate the practicability of NaNb_13_O_33_, the NaNb_13_O_33_ working electrode is coupled with a LiFePO_4_ cathode. At 25 mA g^−1^, the LiFePO_4_/NaNb_13_O_33_ full cell shows a reversible capacity of 186.8 mAh g^−1^ (Figure 2i). At 125, 250, 500, and 1250 mA g^−1^, 154.4, 134.6, 110.3, and 75.0 mAh g^−1^ remain, respectively (Figure 2j,k). It exhibits 95.3% capacity retention over 1000 cycles at 500 mA g^−1^ (Figure 2l). All the half‐ and full‐cell electrochemical data clearly reveal that NaNb_13_O_33_ can be a practical Li^+^‐storage material with a proper operating potential, high first‐cycle Coulombic efficiency, large reversible capacity, outstanding rate performance, excellent cyclic stability, and good low‐temperature electrochemical properties, which is especially suitable for the high‐performance LIBs used in electric logistics vehicles.

### 2.3 Redox Mechanism and Electrochemical Kinetics

**Figure 3:**
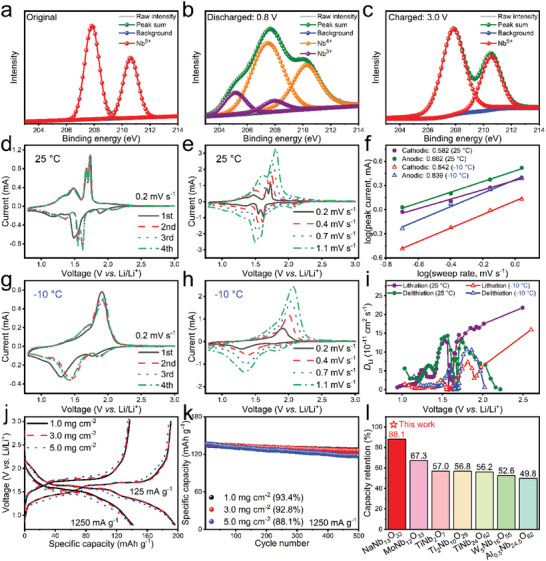
Redox mechanism and electrochemical kinetics of NaNb_13_O_33_. Ex situ Nb‐3*d* XPS spectra: a) original, b) discharged, and c) charged states. CV curves of half cells at 0.2 mV s^−1^: d) 25 and g) −10 °C. CV curve of half cells at various sweep rates: e) 25 and h) −10 °C. f) Calculations of *b*‐values for half cells at 25 and −10 °C. i) Variations in Li^+^ diffusion coefficients of NaNb_13_O_33_ at 25 and −10 °C. j) GCD curves of half cells with various active‐material loadings at 125 and 1250 mA g^−1^. k) Cyclic stability of half cells with various active‐material loadings at 1250 mA g^−1^. l) Capacity retention of NaNb_13_O_33_ with large active‐material loading of ≈5.0 mg cm^−2^ at 1250 mA g^−1^ after 500 cycles, and its comparison with that of typical shear ReO_3_‐type niobates.

The survey XPS spectrum in Figure S7 (Supporting Information) clearly shows the existence of Na, Nb, and O elements in NaNb_13_O_33_. Before discharge, the Nb‐3*d* spectrum consists of a Nb‐3*d*_5/2_ and Nb‐3*d*_3/2_ doublet at 207.8 and 210.5 eV (**Figure** **3**a), suggesting that the valences state of the Nb element is +5.^[^ ^16a^ ^]^ After full discharge (Figure 3b), the doublet at 207.5 and 210.2 eV corresponds to Nb‐3*d*_5/2_ and Nb‐3*d*_3/2_ of Nb^4+^, whereas the doublet at 205.2 and 207.9 eV is attributed to Nb^3+^.^[^ ^20^ ^]^ After full charge (Figure 3c), the Nb valence almost fully recovers +5, indicating the excellent Nb^5+^↔Nb^3+^ reversibility in NaNb_13_O_33_.

The Cyclic voltammogram (CV) experiments at different temperatures are performed on the half cells, revealing the redox mechanism of NaNb_13_O_33_. At 25 °C and 0.2 mV s^−1^ (Figure 3d), the first‐cycle CV curves are slightly different from that in the subsequent cycle, which can be rooted in the formation of thin SEI films and the irreversible polarization during the first cycle.^[^ ^21^ ^]^ After the first cycle, however, the CV curves display good repeatability. The second cycle exist three obvious CV‐peak pairs centered at 1.62/1.73, 1.55/1.67, and 1.36/1.47 V, respectively. Both the first and second pairs could be assigned to the Nb^4+^/Nb^5+^ redox reaction.^[^ ^22^ ^]^ This peak split for Nb^4+^/Nb^5+^ could be ascribed to the Li^+^ insertion into different lattice sites. The third pair could correspond to the Nb^3+^/Nb^4+^ redox couple.^[^ ^22^ ^]^ At −10 °C (Figure 3g), the positions of the cathodic/anodic peaks for Nb^4+^/Nb^5+^ shift to 1.29/1.91 V in the first cycle, indicating increased electrode polarization than that at 25 °C, as expected. The high‐potential peaks do not obviously split and the low‐potential peaks become weak, which could be rooted in the smaller capacity at the low temperature.

At different sweep rates and temperatures (Figure 3e,h), the CV‐peak current (*I*) keeps increasing with the sweep rate (*v*), which can conform to the equation of *I* = *av* ^b^,^[^ ^23^ ^]^ in which *a* and *b* (0.5≤*b*≤1) are adjustable parameters. A larger *b* value indicates more significant capacitive behavior, which enables faster charge transport because the capacitive behavior is not determined by solid‐state diffusion.^[^ ^24^ ^]^ The *b* values for the intensive anodic and cathodic peaks are respectively determined to be 0.582 and 0.662 at 25 °C, and those at −10 °C remarkably increase to 0.839 and 0.842 (Figure 3f), suggesting that the intercalation‐pseudocapacitive behavior in NaNb_13_O_33_ is significant at 25 °C and becomes much more significant at −10 °C due to the undoubtedly slower low‐temperature Li^+^ diffusivity (as described below). This phenomenon can be attributed to its very large interlayer spacing,^[^ ^16a^ ^]^ similar to the case of T‐Nb_2_O_5_.^[^ ^23b^ ^]^ Undoubtedly, the existence of the intercalation‐pseudocapacitive behavior in NaNb_13_O_33_ benefits its electrochemical kinetics and Li^+^‐storage properties (especially at the low temperature).

The Li^+^ apparent diffusion coefficients (*D*_Li_) of NaNb_13_O_33_ at different temperatures are calculated through different methods.^[^ ^25^ ^]^ At 25 °C, its average *D*_Li_ values calculated from the galvanostatic intermittent titration technique (GITT) data reach 6.00 × 10^−11^ and 5.70 × 10^−11^ cm^2^ s^−1^ during lithiation and delithiation, respectively (Figure 3i, calculation details in Supporting Information), which are among the best results in the previously‐reported shear ReO_3_‐type niobates (Table S5, Supporting Information). Although the temperature significantly drops to −10 °C, its *D*_Li_ values still retain 1.60 × 10 (lithiation) and 2.30 × 10^−11^ cm^2^ s^−1^ (delithiation). It is noteworthy that this Li^+^ diffusivity of NaNb_13_O_33_ at such low temperature is even faster than that of most shear ReO_3_‐type niobates at 25 °C (Table S5, Supporting Information). Similar Li^+^‐diffusivity results are achieved through the CV method (Figure S9, calculation details in Supporting Information). The DFT calculations reveal 3D Li^+^ transport pathways for fast Li^+^ transport with a maximum energy barrier of only ≈0.5 eV (Figure S10, calculation details in Supporting Information). Clearly, the very fast Li^+^ diffusivity in NaNb_13_O_33_ undoubtedly arises from its open shear ReO_3_‐type interlayered structure with the very large interlayer spacing.^[^ ^16a^ ^]^ In summary, the intrinsically fast Li^+^ diffusivity and significant intercalation‐pseudocapacitive behavior in NaNb_13_O_33_ work together, achieving its prominent rate performance at 25 and −10 °C.

With increasing the active‐material loading of the working electrodes respectively by two and four times, the reversible capacity of NaNb_13_O_33_ is not obviously decreased at both low (125 mA g^−1^) and high (1250 mA g^−1^) current rates (Figure 3j). The thick electrode (5.0 mg cm^−2^) shows much higher rate performance than commercial graphite (Figure S11, Supporting Information), and is still capable of delivering excellent cyclic stability with 88.1% capacity retention at 1250 mA g^−1^ after 500 cycles (Figure 3k), even though the particle sizes of NaNb_13_O_33_ are on the order of micrometers. Surprisingly, this retention percentage of NaNb_13_O_33_ is significantly higher than that of niobates with similar active‐material loadings and micron‐sized particles (Figure 3l; Figure S12, Supporting Information), such as TiNb_2_O_7_ (57.0%), Ti_2_Nb_10_O_29_ (56.8%), TiNb_24_O_62_ (56.2%), MoNb_12_O_33_ (67.3%), W_5_Nb_16_O_55_ (52.6%), and Al_0.5_Nb_24.5_O_62_ (49.8%). In addition, this cyclic stability of NaNb_13_O_33_ is also better than that of T‐Nb_2_O_5_ (Figure S13, Supporting Information), and even comparable to that of “zero‐strain” Li_4_Ti_5_O_12_ (Figure S14, Supporting Information). Therefore, the superior kinetics and practicability of NaNb_13_O_33_ are further confirmed.

### 2.4 Crystal‐Structure Evolutions

**Figure 4:**
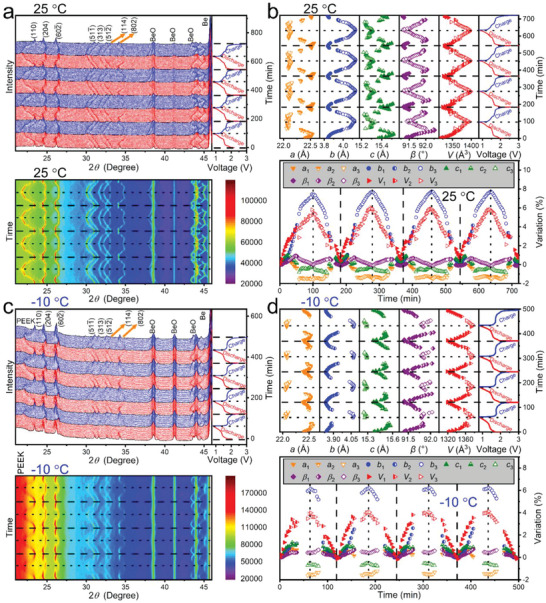
Temperature‐dependent crystal‐structure evolutions of NaNb_13_O_33_. a, c) Pristine in situ XRD patterns with GCD curves and contour in situ XRD patterns of in situ half cells at 25 and −10 °C (first four cycles). b, d) Lattice‐constant variations of NaNb_13_O_33_ at 25 and −10 °C (first four cycles). Solid, half‐solid, and hollow symbols correspond to original phase, transformed phase after first phase transformation, and transformed phase after second phase transformation, respectively.

**Figure** **4**a shows the first‐four‐cycle pristine and contour in situ XRD patterns collected at 25 °C and 125 mA g^−1^. During the first lithiation, NaNb_13_O_33_ undergoes three obvious phase transformations when lithiation to ≈1.6, ≈1.5, and ≈1.3 V. Before the first phase transformation, the (110), (204), and (60$\bar{2}$) peaks shift toward smaller angles, and their intensity changes are not obvious. During the first phase transformation, the (110) peak shifts from 23.3° to 23.2°, whereas the (204) and (60$\bar{2}$) peaks shift toward larger angles, and their peak intensities significantly decrease. Between the first and second phase transformations, the (110) peak continues shifting toward smaller angles, whereas the (204) and (60$\bar{2}$) peaks continue shifting toward larger angles, and their peak intensities slowly decrease. During the second phase transformation, the (110) peak shifts from 22.6° to 22.2° and the (60$\bar{2}$) peak shifts from 26.2° to 26.4°, whereas the (204) peak shifts slightly toward larger angles, and their peak intensities significantly decrease. Between the second and third phase transformations, the (110) and (204) peaks shift toward smaller angles, whereas the (60$\bar{2}$) peak slowly shifts toward larger angles, and their peak intensities first increase and then decrease gradually. During the third phase transformation, the (110) and (204) peaks continue shifting toward smaller angles, whereas the (60$\bar{2}$) peak continues shifting toward larger angles, and their peak widths and intensities significantly increase and decrease, respectively.^[^ ^26^ ^]^

These complicated evolutions of the peaks significantly reverse during the subsequent delithiation. However, the delithiation and lithiation processes are not completely reversible (dotted squares in Figure S15, Supporting Information) because it is possible that the Li^+^‐extraction sequence may not fully reverse the Li^+^‐insertion one. The (110) peak‐intensity change in the second lithiation is slightly different from that in the first lithiation during the first phase transformation (dotted circles in Figure S15, Supporting Information), which matches with the CV results. The peak evolutions in the following cycles, however, are almost the same as those in the second cycle, confirming the superior crystal‐structure stability of the intercalating NaNb_13_O_33_.

During lithiation, the Rietveld‐refined *a*, *b*, *c*, and *β* values of NaNb_13_O_33_ gradually increase before the first phase transformation (Figure 4b). As a result, the *V* value increases by 2.68%. The first transformed phase reveals slightly smaller *a*, *c*, and *V* (0.41% shrinkage) values, and slightly larger *b* and *β* values. Between the first and second phase transformations, the *a* and *c* values decrease gradually, and the *b* and *β* values increase gradually. Thus, the *V* value increases by 1.63%. The second transformed phase exhibits slightly smaller *a*, *c, β*, and *V* (0.76% shrinkage) values, and a slightly larger *b* value. Between the second and third phase transformations, the *a*, *b*, and *c* values increase gradually, and the *β* value decreases gradually. Consequently, the *V* value increases by 1.15%. The third transformed phase shows slightly larger *a*, *b*, and *c* values, and a slightly smaller *β* value. Therefore, the *V* value continues increasing until 0.8 V (1.73%). Importantly, the lattice‐constant variations during each phase transformation are small, thereby allowing the achievement of the high rate performance, which is similar to the case of Li_4_Ti_5_O_12_ with Li_4_Ti_5_O_12_↔Li_7_Ti_5_O_12_ phase transformation.^[^ ^5^ ^]^ The lattice‐constant variations during delithiation do not fully reverse those during lithiation, and the *a*‐, *c*‐, and *β*‐value variations during the first phase transformation in the last three lithiation processes are slightly different from those in the first lithiation process, matching well with the peak variations. The total *V*‐value variation of NaNb_13_O_33_ is limited to 6.02% at 0.8 V. These lattice‐constant variations are verified by the ex situ high‐resolution transmission electron microscopy (HRTEM) results (Figure S16, Supporting Information). This maximum unit‐cell‐volume variation of NaNb_13_O_33_ is remarkably smaller than that of the previously‐reported shear ReO_3_‐type niobates (Table S6, Supporting Information), undoubtedly leading to the excellent cyclic stability of NaNb_13_O_33_.

At −10 °C, however, NaNb_13_O_33_ only undergoes two obvious phase transformations respectively at ≈1.4 and ≈1.3 V during lithiation (Figure 4c). The third phase transformation does not occur (dotted circles in Figure S17, Supporting Information), attributed to the smaller inserted‐Li^+^ amount at −10 °C. In addition, the second phase transformation process is prolonged, as revealed by the dotted rectangles in Figure S17 (Supporting Information), which could be ascribed to the slower Li^+^ diffusivity at −10 °C. The *a*‐, *c*‐, and *β*‐value variations are similar to those at 25 °C, whereas the *b*‐ and *V*‐value changes are significantly smaller (Figure 4d). The maximum *V*‐value variation of NaNb_13_O_33_ is significantly limited to only 4.12% at −10 °C. This *V*‐value decrease is reasonable owing to the smaller Li^+^‐storage amount at −10 °C, and undoubted results in the even better cyclic stability at −10 °C.

**Figure 5:**
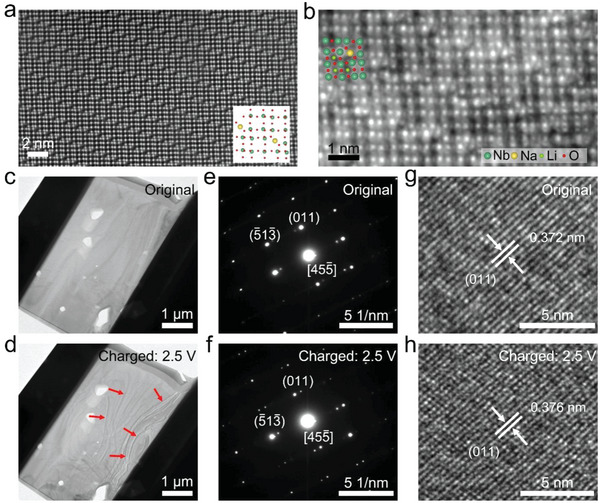
STEM and in situ TEM characterizations. a) HAADF image and b) iDPC image of lithiated NaNb_13_O_33_ (0.8 V) with inverted image contrast, revealing Li^+^‐insertion positions. In situ TEM images of NaNb_13_O_33_ at c) original and d) charged (2.5 V) states. Strain fringes induced by Li^+^ insertion are highlighted by red arrows. In situ SAED patterns of NaNb_13_O_33_ at e) original and f) charged (2.5 V) states. In situ HRTEM images of NaNb_13_O_33_ at g) original and h) charged (2.5 V) states.

The HAADF‐STEM image (**Figure** **5**a) demonstrates that the synthesized NaNb_13_O_33_ owns excellent structural stability during lithiation. The integrated differential phase contrast (iDPC)‐STEM image (Figure 5b) of the lithiated NaNb_13_O_33_ reveals the inserted Li^+^ ions, as highlighted by green balls. Additionally, the in situ XRD results (Figure 4b,d) indicate that the *V*‐value variation is mainly determined by the *b*‐value variation. It can therefore be concluded that the external Li^+^ ions mainly insert into the interstices between the interlayers of NaNb_13_O_33_.

To directly observe the lithiation process of NaNb_13_O_33_, in situ transmission electron microscopy (in situ TEM) is used based on a micron‐sized solid‐state cell,^[^ ^27^ ^]^ which is built by sandwiching the Li_6.4_La_3_Zr_1.4_Ta_6_O_12_ (LLZO) solid‐state electrolyte between the NaNb_13_O_33_ anode and LiFePO_4_ cathode. The Li^+^ insertion into NaNb_13_O_33_ results in strain fringes, which exhibit clear movement (Figure 5c,d; Supporting Video). However, the volume and morphology variations are very small, verifying the intercalation nature of NaNb_13_O_33_ with very limited unit‐cell‐volume variations. Moreover, the in situ SAED patterns (Figure 5e,f) and in situ HRTEM images (Figure 5g,h) recorded at original and charged (2.5 V) states show very limited differences, which further confirms the small lattice‐constant variations.

## 3 Conclusion

NaNb_13_O_33_ micron‐sized particles are developed as a practical anode material with comprehensively good electrochemical properties and broad temperature adaptability. The largest interlayer spacing (3.8484 Å) in this new shear ReO_3_‐type niobate greatly benefits its rate performance, cyclic stability, and low‐temperature electrochemical properties. The active Nb^5+^↔Nb^3+^ redox reaction in NaNb_13_O_33_ is highly reversible, allowing its large reversible capacities of 221.6 (25 °C) and 176.1 mAh g^−1^ (−10 °C), high first‐cycle Coulombic efficiencies of 92.9% (25 °C) and 96.1% (−10 °C), and safe operating potentials of ≈1.52 (25 °C) and ≈1.59 V (−10 °C) at 25 mA g^−1^. Its large interlayer spacing allows notable intercalation‐pseudocapacitive behavior and very fast Li^+^ diffusivity. These advantages are significantly beneficial for its rate performance, which shows a large 1250 to 125 mA g^−1^ capacity percentage of 71.8% and 2500 to 1250 mA g^−1^ percentage of 63.2% at 25 °C, and a 1250 to 125 mA g^−1^ percentage of 65.6% at −10 °C. The stable shear ReO_3_‐type structure with the large interlayer spacing limits the maximum unit‐cell‐volume expansions to 6.02% (25 °C) and 4.12% (−10 °C) after the majority of the external Li^+^ ions insert into the interstices between the interlayers, enabling desirable cyclic stability with high capacity retentions of 87.9% (25 °C and 2500 mA g^−1^) and 105.4% (−10 °C and 1250 mA g^−1^) after 5000 cycles. The insight gained here can promote the exploration of fast‐charging, long‐life, and low‐temperature friendly energy‐storage materials.

## Conflict of Interest

The authors declare no conflict of interest.

## Supporting information

Supporting Information

Supplemental Video 1

## Data Availability

The data that support the findings of this study are available from the corresponding author upon reasonable request
