# Supplementary material for: Sodium Niobate with a Large Interlayer Spacing: A Fast‐Charging, Long‐Life, and Low‐Temperature Friendly Lithium‐Storage Material
Source: Adv Sci (Weinh). 2023 Apr 29;10(20):2300583. doi: 10.1002/advs.202300583 (PMC10369234; doi:10.1002/advs.202300583)
Supplement: Supplementary file 1 — Supporting Information [file ADVS-10-2300583-s002.pdf]

ADVANCED SCIENCE
Open Access

Supporting Information

for *Adv. Sci.*, DOI 10.1002/advs.202300583

Sodium Niobate with a Large Interlayer Spacing: A Fast-Charging, Long-Life, and Low-Temperature Friendly Lithium-Storage Material

Jiazhe Gao, Liting Yang, Cihui Huang, Guisheng Liang, Yi Lei, Songjie Li, Wenze Wang, Yinjun Ou, Shangfu Gao, Xuehua Liu, Yifeng Cheng, Jincang Zhang, Zhongzhu Liu, Aiming Guo, Robson Monteiro, Luanna Parreira, Rogerio Ribas, Chunfu Lin, Limin Wu* and Renchao Che**

Supporting Information

Sodium Niobate with a Large Interlayer Spacing: A Fast-Charging, Long-Life, and Low-Temperature Friendly Lithium-Storage Material

Jiazhe Gao, Liting Yang, Cihui Huang, Guisheng Liang, Yi Lei, Songjie Li, Wenze Wang, Yinjun Ou, Shangfu Gao, Xuehua Liu, Yifeng Cheng, Jincang Zhang, Zhongzhu Liu, Aiming Guo, Robson Monteiro, Luanna Parreira, Rogerio Ribas, Chunfu Lin, Limin Wu*, and Renchao Che**

Experimental Section

Material Preparation: $NaNb_{13}O_{33}$ was fabricated through a two-step solid-state reaction process. First, sodium Na_2CO_3 (Macklin, 99.5%) and Nb_2O_5 (AD 8758, Companhia Brasileira de Metalurgia e Mineração (CBMM)) with a 1 : 1 molar ratio were milled in a high-energy ball miller (SPEX 8000M) for 1 h. After sintering the milled powder at 1100 °C for 4 h in air, $NaNbO_3$ micron-sized particles were obtained. Then, $NaNbO_3$ and Nb_2O_5 with a 1 : 6 molar ratio were milled in the high-energy ball miller for 1h. Finally, the milled powder was calcinated at 1100 °C for 4 h in air, forming $NaNb_{13}O_{33}$ micron-sized particles.

Material Characterizations: The powder X-ray diffraction (XRD) pattern of $NaNb_{13}O_{33}$ was collected using X-ray diffractometry (Rigaku Ultima IV), and Rietveld-refined using the free General Structure Analysis System (GSAS) software.[S1] The particle morphology and microstructure were recorded using field emission scanning electron microscopy (FESEM, JEOL JSM-7800F) equipped with energy-dispersive X-ray spectroscopy (EDX, OXFORD X-Max), and high-resolution transmission electron microscopy (HRTEM, JEOL JEM-2100F). The cation valences were determined by X-ray photoelectron spectroscopy (XPS, PHI5000 Versaprobe III). The high-angle annular dark-field (HAADF) and integrated differential phase contrast (iDPC) scanning TEM (STEM) images were recorded using dual spherical aberration-corrected electron microscopy (ThermoFisher Scientific Titan). The specific surface area was obtained using a surface area analyzer (ASAP 2460) and the Brunauer−Emmett−Teller (BET) model. X-ray fluorescence (XRF) spectrometer (ARL ADVANT'X IntelliPowerTM 3600) was used to determine the exact Na : Nb molar ratio in $NaNb_{13}O_{33}$.

Electrochemical Tests: CR2032-type coin cells were assembled in a glove box for examining the galvanostatic charge–discharge (GCD) properties of $NaNb_{13}O_{33}$ on an automatic battery testing system (Neware CT-3008) at 25 and −10 °C, which were controlled in a temperature-variable cryostat system (Linpin LRHS-101C). Li foils were used as anodes and counter electrodes in the half cells. The working electrodes were fabricated by coating homogenous mixture composed of $NaNb_{13}O_{33}$, Super-P® conductive carbon, and polyvinylidene fluoride (8 : 1 : 1 in mass) on Cu current collectors, with an active-material loading of 1.0/3.0/5.0 mg cm^{-2}. The electrolyte was composed of 1 M $LiPF_6$ in an ethylene carbonate, diethylene carbonate, and dimethyl carbonate mixed solvent (1 : 1 : 1 in volume). Glass fibers (Whatman GF/D-1823) were used as separators. Cyclic voltammogram (CV) curves were recorded on an electrochemical workstation (Gamry Interface 1010E). The half cells were examined within 0.8–3.0 V *vs*. Li/Li^+.

$NaNb_{13}O_{33}$ and $LiFePO_4$ (P198, Shenzhen BTR New Energy Materials) were respectively employed as anode and cathode materials for the full cells, with an N/P ratio of 0.93 (anode-limited configuration). Al foils were used as the current collectors for the $LiFePO_4$ cathodes. Celgard® 2325 microporous polypropylene films served as the separators. The full cells were examined within 1.0–2.5 V, and the specific capacities were calculated based on the mass of $NaNb_{13}O_{33}$.

In-Situ Examinations: The assembly of the *in-situ* XRD cells for 25 °C was the same as that in half cells except that Be plates served as not only the current collectors but also the low-X-ray-penetration windows in a commercial module (LIB-XRD, Beijing Scistar Technology).[S2] In addition, to keep the working temperature at −10 °C, a temperature-control unit and polyetheretherketone (PEEK) dome were integrated into another *in-situ* cell (XRD-LHTXRD-LN, Beijing Scistar Technology).[S2] The *in-situ* XRD experiments at these two temperatures were performed during GCD at 125 mA g^{-1}.

A micron-sized cell setup within an electrical biasing TEM holder (Gatan instruments), which was loaded on the transmission electron microscope (JEOL JEM-2100F), was used to directly observe the Li^+ insertion into $NaNb_{13}O_{33}$.[S3] Li^+ ions in a $LiFePO_4$ cathode were extracted by a voltage bias from the electrochemical workstation (Gamry Interface 1010E), then passed through $Li_{6.4}La_3Zr_{1.4}Ta_6O_{12}$ (LLZO) solid-state electrolyte, and finally inserted into a $NaNb_{13}O_{33}$ anode.

DFT Calculations: The Vienna Ab-initio Simulation Package (VASP) package with density functional theory (DFT) as the theoretical framework was used for the calculations.[S4,S5] The electronic exchange-correlation energy was determined using the Perdew–Burke–Ernzerhof

functional within the generalized gradient approximation.[S6] The cutoff energy was set to 400 eV, and the convergence criteria were 1.0×10^{-3} eV for energy and 0.01 eV $Å^{-1}$ for stress. The unit cell was modeled with a Monhkorst–Pack *k*-point mesh of 1×4×1. The climbing image nudged elastic band (CI-NEB) method was employed to locate the transition states for Li^+ diffusion.[S7,S8] The crystal structure of $NaNb_{13}O_{33}$ was optimized using a bulk structure consisting of two $NaNb_{13}O_{33}$ units ($[NaNb_{13}O_{33}]_2$). The geometry structures and binding energies (E_b) of $Li_x[NaNb_{13}O_{33}]_2$ were calculated at various Li^+ concentrations. The binding energy of $Li_x[NaNb_{13}O_{33}]_2$ was computed using **Equation S1**:

$$E_b = E_T - E_R - xE_{Li} \tag{S1}$$

where E_T and E_R were the total energy of $Li_x[NaNb_{13}O_{33}]_2$ and $[NaNb_{13}O_{33}]_2$, respectively; E_{Li} was obtained from Li metal.

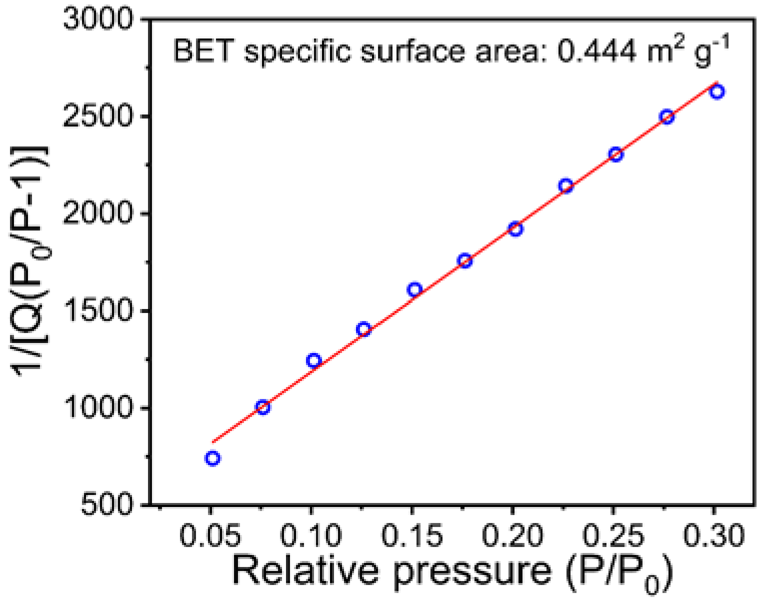

Figure S1. BET specific surface area plot of $NaNb_{13}O_{33}$.

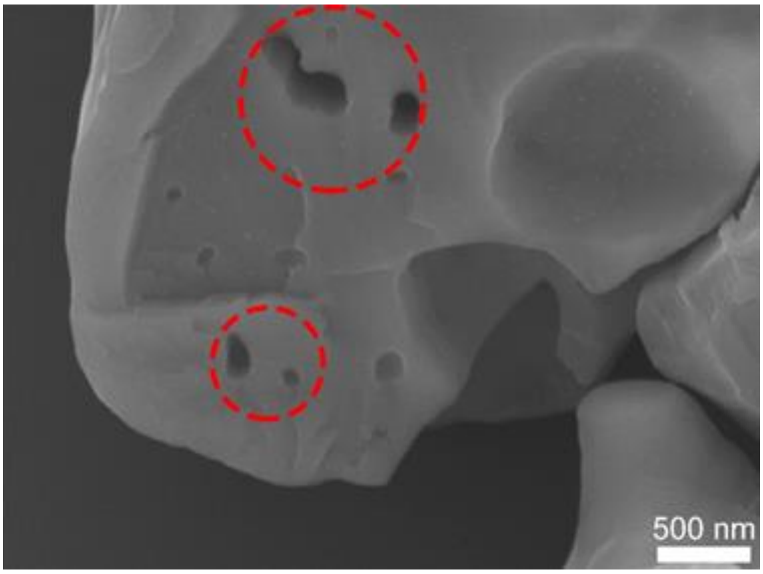

Figure S2. FESEM image of $NaNb_{13}O_{33}$ showing that abundant nanopores exist in $NaNb_{13}O_{33}$ particle (several nanopores are marked by dotted cycles).

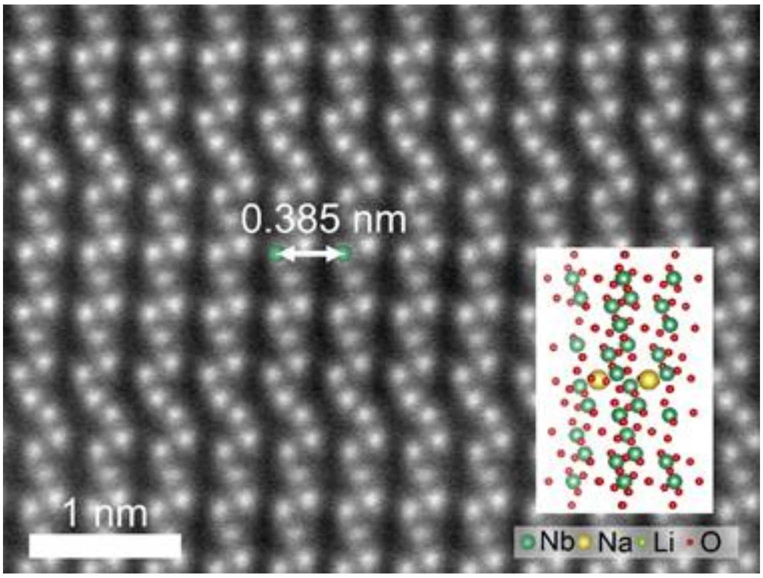

Figure S3. HAADF-STEM image of $NaNb_{13}O_{33}$.

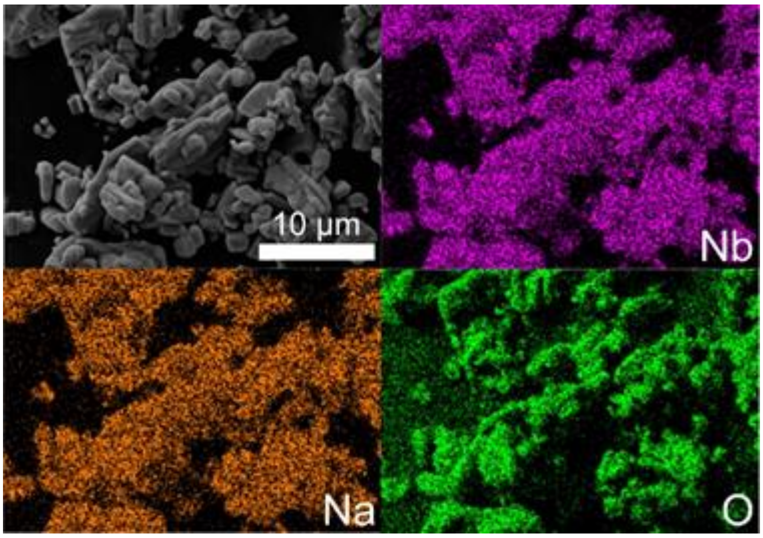

Figure S4. EDX mapping images of $NaNb_{13}O_{33}$.

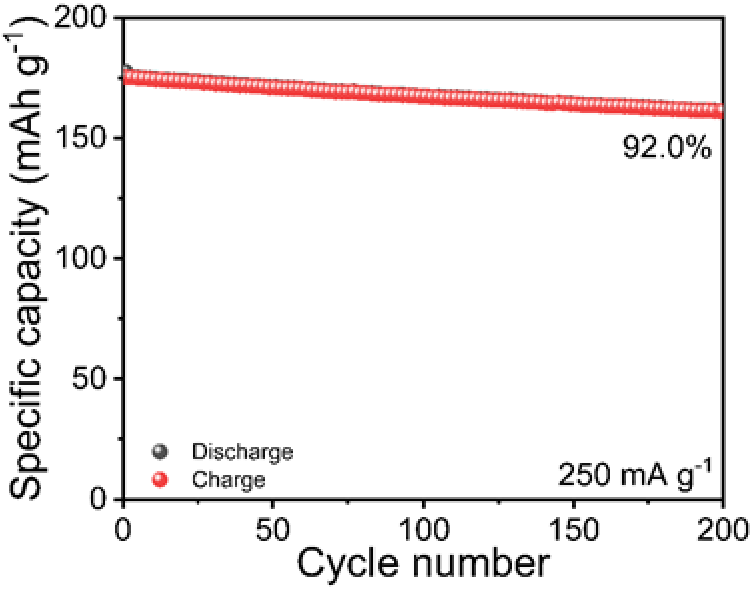

Figure S5. Cyclic stability of $NaNb_{13}O_{33}$/Li half cell after 200 cycles at 250 mA g^{-1}.

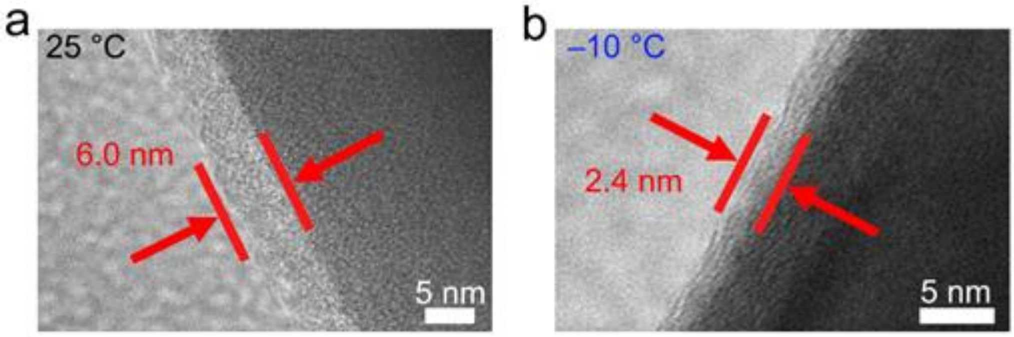

Figure S6. TEM images of $NaNb_{13}O_{33}$ samples showing SEI films after first lithiation to 0.8 V at a) 25 and b) −10 °C.

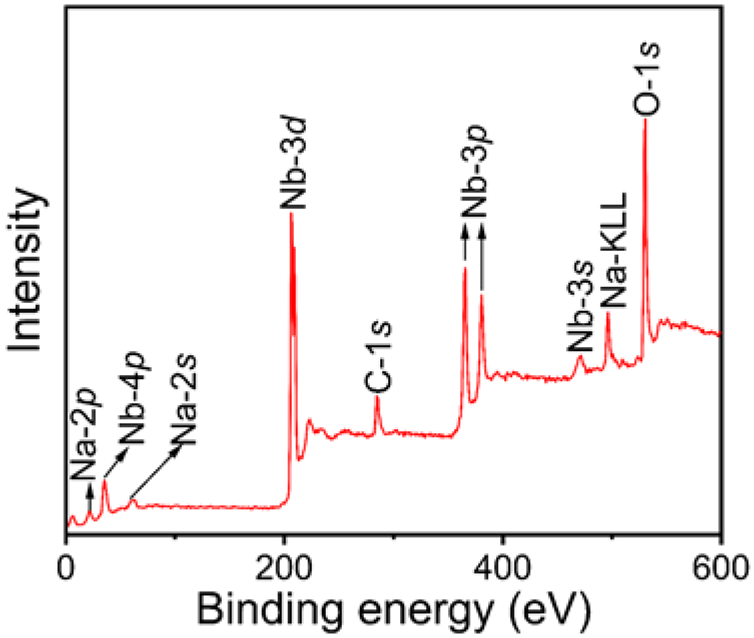

Figure S7. XPS survey spectrum of $NaNb_{13}O_{33}$.

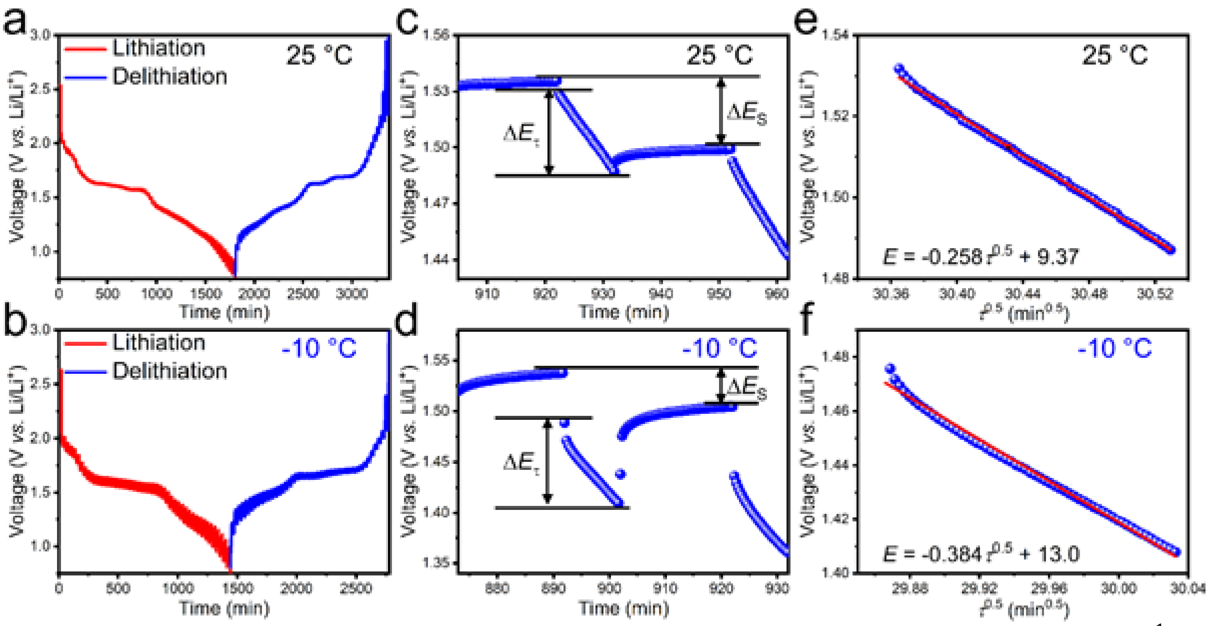

Figure S8. GITT lithiation–delithiation profiles of $NaNb_{13}O_{33}$/Li half cells at 25 mA g^{-1}: a) 25 and b) −10 °C. E *vs.* t profiles for a single step in GITT experiment of $NaNb_{13}O_{33}$: c) 25 and d) −10 °C. Linear behavior of E *vs.* $\tau^{0.5}$ relationship during a typical titration in $NaNb_{13}O_{33}$: e) 25 and f) −10 °C.

Calculation of apparent Li^{+} diffusion coefficients of $NaNb_{13}O_{33}$ by GITT

The GITT experiments are employed to investigate the Li^{+} diffusion behavior in the $NaNb_{13}O_{33}$ lattice at 25 and −10 °C. **Figure S8a** and **Figure S8b** respectively present the GITT profiles of the $NaNb_{13}O_{33}$/Li half cells at 25 and −10 °C. A typical single step of GITT is clearly seen from **Figure S8c**/**Figure S8d**. On the basis of the Fick's second law, the apparent Li^{+} diffusion coefficients (D_{Li}) of $NaNb_{13}O_{33}$ can be determined by using **Equation S2**:[S9]

$$D_{\mathrm{Li}} = \frac{4}{\pi}\left(\frac{m_{\mathrm{b}}V_{\mathrm{m}}}{M_{\mathrm{b}}S}\right)^2\left(\frac{\Delta E_{\mathrm{s}}}{\tau\left(dE_{\tau}/d\sqrt{\tau}\right)}\right)^2 \qquad \left(\tau \ll \frac{L^2}{D_{\mathrm{Li}}}\right) \tag{S2}$$

where, M_{b} is the molar mass of $NaNb_{13}O_{33}$, V_{m} is the molar volume of $NaNb_{13}O_{33}$, m_{b} is the mass of $NaNb_{13}O_{33}$, S is the electrode surface area, τ is the pulse duration time, L is the electrode thickness, and ΔE_{s} and ΔE_{τ} respectively represent the change in the equilibrium potential and the change in potential during the current pulse, which can be gained from the GITT profiles (**Figure S8c**, **d**). As the potential during a single titration delivers a linear relationship with $\tau^{0.5}$ (**Figure S8e**/**Figure S8f**), **Equation S2** can be simplified as **Equation S3**:

$$D_{\mathrm{Li}} = \frac{4}{\pi\tau}\left(\frac{m_{\mathrm{b}}V_{\mathrm{m}}}{M_{\mathrm{b}}S}\right)^2\left(\frac{\Delta E_{\mathrm{s}}}{\Delta E_{\tau}}\right)^2 \qquad \left(\tau \ll \frac{L^2}{D_{\mathrm{Li}}}\right) \tag{S3}$$

Based on **Equation S3**, the apparent Li^{+} diffusion coefficients of $NaNb_{13}O_{33}$ during different discharge/charge states are obtained at 25 and −10 °C, and displayed in **Figure 3i**.

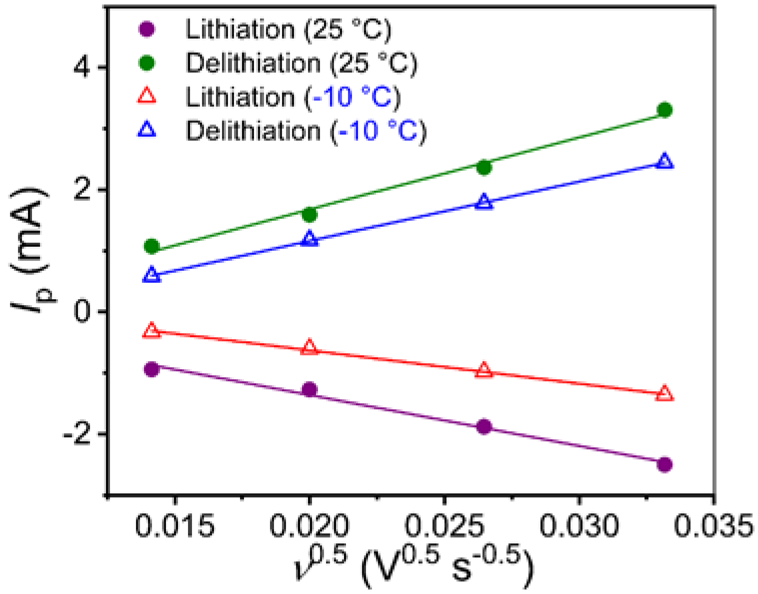

Figure S9. Relationship between peak current (I_p) and square root of sweep rate ($v^{0.5}$) for intensive cathodic/anodic peaks of $NaNb_{13}O_{33}$/Li half cells at 25 and −10 °C.

Calculation of apparent Li^+ diffusion coefficients of $NaNb_{13}O_{33}$ by CV

The apparent Li^+ diffusion coefficients of $NaNb_{13}O_{33}$ during the phase transformation at 25 and −10 °C are also determined from its CV data at different sweep rates (**Figure 3e**, **h**). It is found that the peak current of the intensive cathodic/anodic reaction I_p is in proportional to the square root of the sweep rate $v^{0.5}$ (**Figure S9**), which shows the linear semi-infinite diffusion in cathodic and anodic processes. Consequently, the Randles–Sevcik equation (**Equation S4**) can be applied,[S9] based on which the Li^+ diffusion coefficient D_{Li} can be calculated.

$$I_p = 2.69 \times 10^5\, Sn^{1.5} C D_{Li}{}^{0.5} v^{0.5} \tag{S4}$$

where, S is the electrode area, n is the charge transfer number, and C is the molar concentration of Li^+ in solid. The D_{Li} values of $NaNb_{13}O_{33}$ during the phase transformation reach 1.80×10^{-11} cm^2 s^{-1} (lithiation) and 3.61×10^{-11} cm^2 s^{-1} (delithiation) at 25 °C, and 7.71×10^{-12} cm^2 s^{-1} (lithiation) and 2.45×10^{-11} cm^2 s^{-1} (delithiation) at −10 °C, which match with the GITT results.

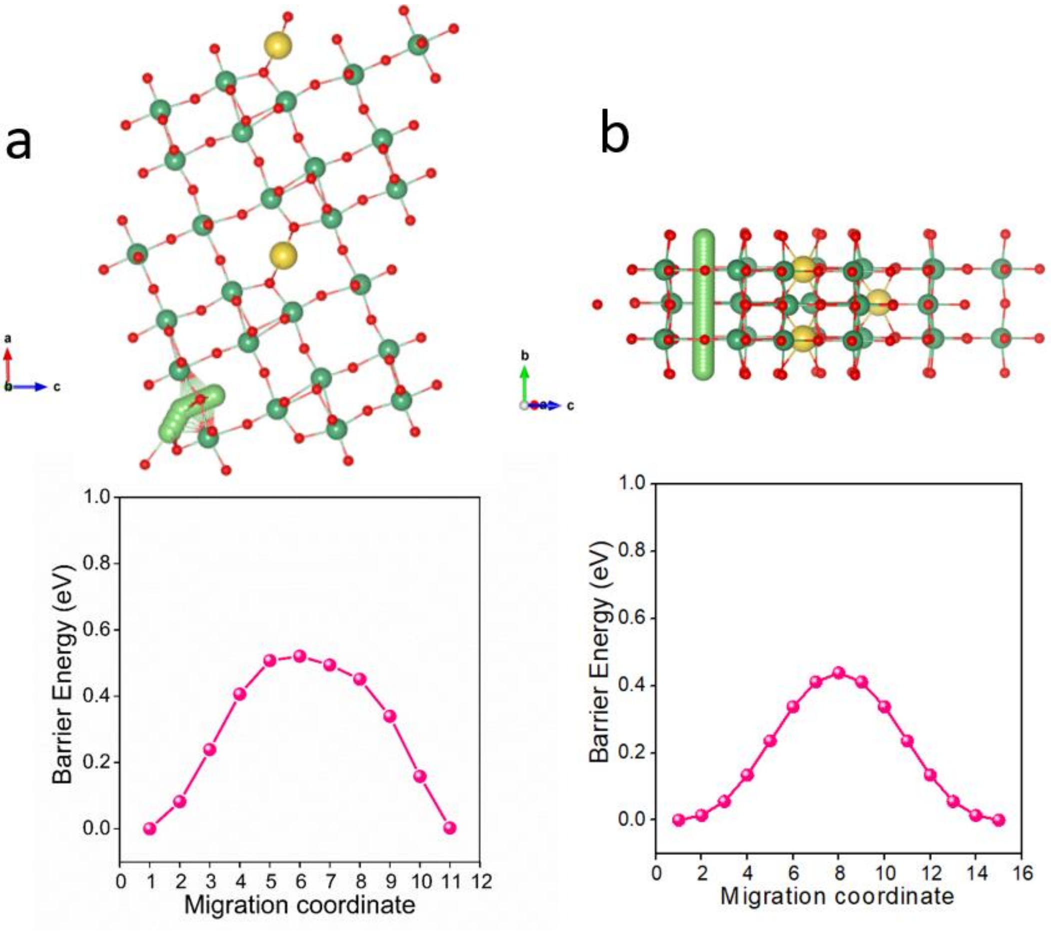

Figure S10. a) Li^+-transport path along interlayer spacing and corresponding energy curve by CI-NEB. b) Li^+-transport path along quadratic channels and corresponding energy curve by CI-NEB.

DFT calculations have been conducted to investigate the Li^+ diffusivity in $NaNb_{13}O_{33}$. The transition states for Li^+ diffusion are located using the climbing image nudged elastic band (CI-NEB) method. The CI-NEB results (**Figure S10**) reveal that Li^+ can transport through the interlayer spacing and quadratic channels with maximum energy barriers of only 0.52 and 0.44 eV, respectively. The small energy-barrier values indicate that these two transport paths are energetically favorable for the Li^+ transport within the framework structure of $NaNb_{13}O_{33}$. Therefore, the resulting three-dimensional and large-sized Li^+ transport pathways are responsible for the fast Li^+ transport in $NaNb_{13}O_{33}$, which significantly contributes to its superior rate performance.

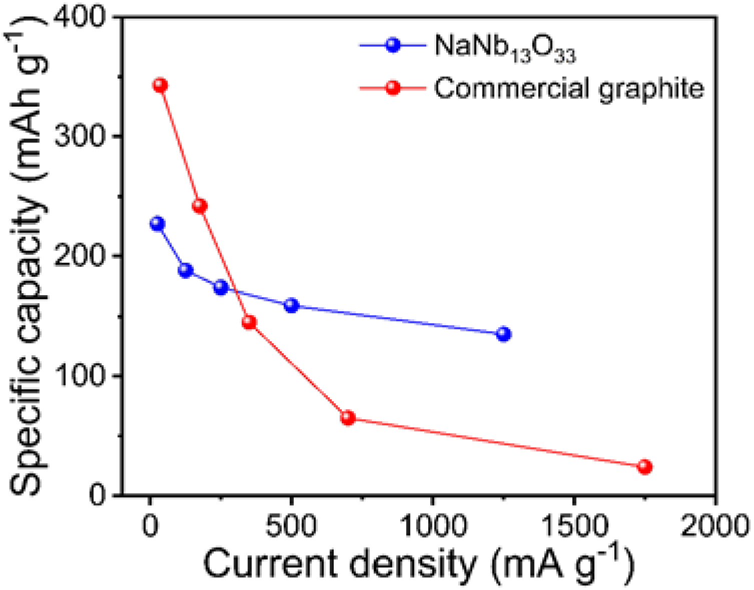

Figure S11. Comparison of rate capability of $NaNb_{13}O_{33}$ and graphite at large active-material loadings of ~5.0 mg cm^{-2}.

The electrochemical properties of the graphite (20019128, Sinopharm Chemical Reagent Co., Ltd.)/Li half cell were tested by the same way with $NaNb_{13}O_{33}$ except that a potential range of 0.01−3.0 V was used.

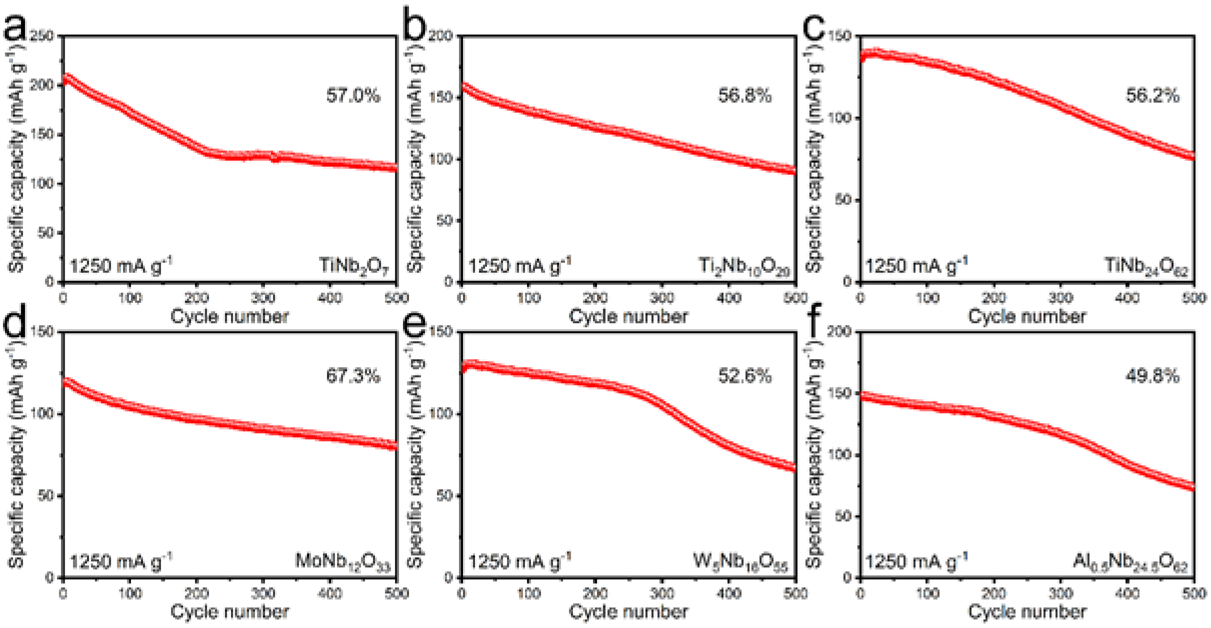

Figure S12. Cyclic stability of shear ReO_3-type niobate anode materials with large active-material loadings of ~5.0 mg cm^{-2} at 1250 mA g^{-1}: a) $TiNb_2O_7$, b) $Ti_2Nb_{10}O_{29}$, c) $TiNb_{24}O_{62}$, d) $MoNb_{12}O_{33}$, e) $W_5Nb_{16}O_{55}$, and f) $Al_{0.5}Nb_{24.5}O_{62}$.

Similar to the $NaNb_{13}O_{33}$ micron-sized particles, the $MoNb_{12}O_{33}$, $W_5Nb_{16}O_{55}$, $TiNb_{24}O_{62}$, $Al_{0.5}Nb_{24.5}O_{62}$, $TiNb_2O_7$ and $Ti_2Nb_{10}O_{29}$ micron-sized particles were prepared by using the solid-state reaction method. For the $MoNb_{12}O_{33}$ micron-sized particles, MoO_3 (Macklin, 99.9%) and Nb_2O_5 (AD 8758, Companhia Brasileira de Metalurgia e Mineração (CBMM)) with a molar ratio of 1 : 6 and the calcination at 900 °C were used. For the $W_5Nb_{16}O_{55}$ micron-sized particles, WO_3 (Macklin, 99.99%) and Nb_2O_5 with a molar ratio of 5 : 8 and the calcination at 1150 °C were used. For the $TiNb_{24}O_{62}$ micron-sized particles, TiO_2 (Aladdin, 99.8%) and Nb_2O_5 with a molar ratio of 1 : 12 and the calcination at 1300 °C were used. For the $Al_{0.5}Nb_{24.5}O_{62}$ micron-sized particles, Al_2O_3 (Macklin, 99.99%) and Nb_2O_5 with a molar ratio of 1 : 49 and the calcination at 1300 °C were used. For the $TiNb_2O_7$ micron-sized particles, TiO_2 (Aladdin, 99.8%) and Nb_2O_5 with a molar ratio of 1 : 1 and the calcination at 1100 °C were used. For the $Ti_2Nb_{10}O_{29}$ micron-sized particles, TiO_2 and Nb_2O_5 with a molar ratio of 1 : 5 and the calcination at 1200 °C were used. In addition, the electrochemical properties of $MoNb_{12}O_{33}$, $W_5Nb_{16}O_{55}$, $TiNb_{24}O_{62}$, $Al_{0.5}Nb_{24.5}O_{62}$, $TiNb_2O_7$ and $Ti_2Nb_{10}O_{29}$ were tested by the same way with $NaNb_{13}O_{33}$.

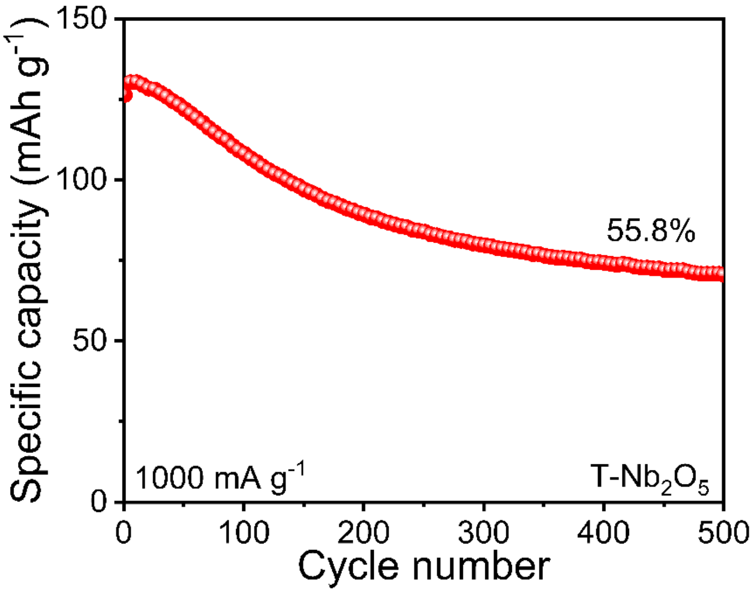

Figure S13. Cyclic stability of T-Nb_2O_5 with a large active-material loading of ~5.0 mg cm^{-2} at 1000 mA g^{-1}.

T-Nb_2O_5 nanoparticles were prepared by using a hydrothermal method. 1.1 g of $C_{10}H_5NbO_{20}$ (Macklin, 98%) was dissolved in 30 mL of deionized water. After stirring at 50 °C for 1 h, the solution was transferred to a 50 mL autoclave, which was then exposed to a 150 °C oven for 12 h for a hydrothermal reaction. The white product after the reaction was collected and washed with deionized water. The obtained paste was vacuum-dried at 80 °C for 10 h and calcined at 700 °C for 4 h in air. The electrochemical properties of T-Nb_2O_5 were tested by the same way with $NaNb_{13}O_{33}$ except that a potential range of 1.0−3.0 V was used.

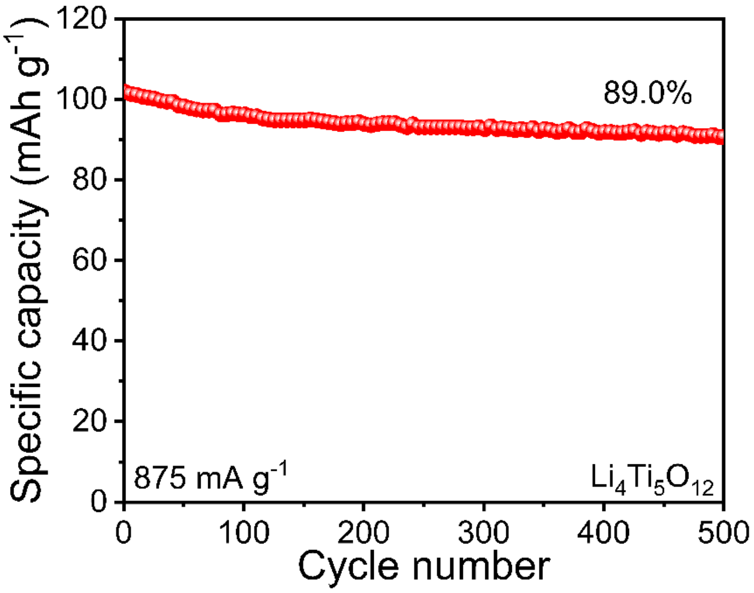

Figure S14. Cyclic stability of $Li_4Ti_5O_{12}$ with a large active-material loading of ~5.0 mg cm^{-2} at 875 mA g^{-1}.

$Li_4Ti_5O_{12}$ was prepared by using the solid-state reaction method, Li_2CO_3 (Macklin, 99.5%) and TiO_2 (Macklin, 99%) with a 2 : 5 molar ratio were milled in the high-energy ball miller for 1 h. After sintering the milled powder at 800 °C for 10 h in air, $Li_4Ti_5O_{12}$ submicron-sized particles were obtained. The electrochemical properties of $Li_4Ti_5O_{12}$ were tested by the same way with $NaNb_{13}O_{33}$ except that a potential range of 1.0−2.5 V was used.

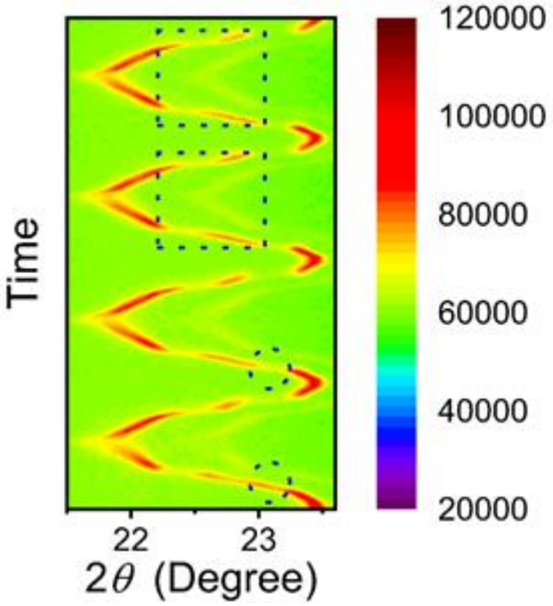

Figure S15. Enlarged contour *in-situ* XRD patterns within 21.5–23.6° at 25 °C.

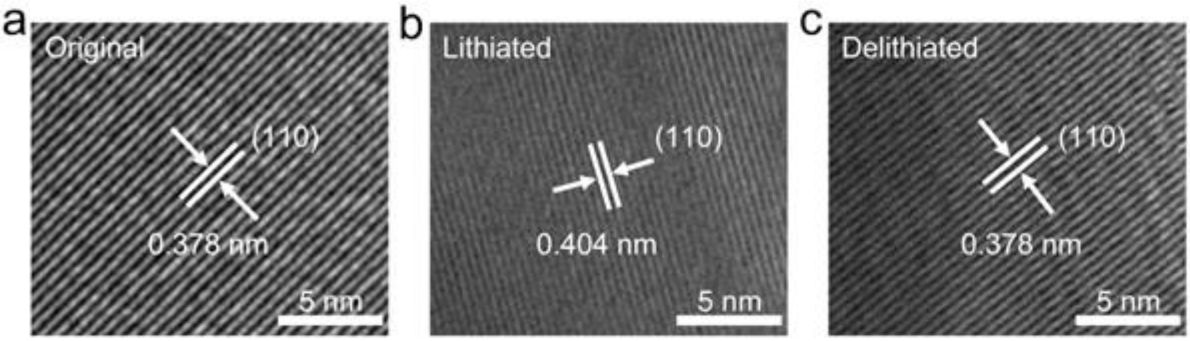

Figure S16. *Ex-situ* HRTEM images of a) original, b) lithiated (0.8 V), and c) delithiated (3.0 V) $NaNb_{13}O_{33}$ samples.

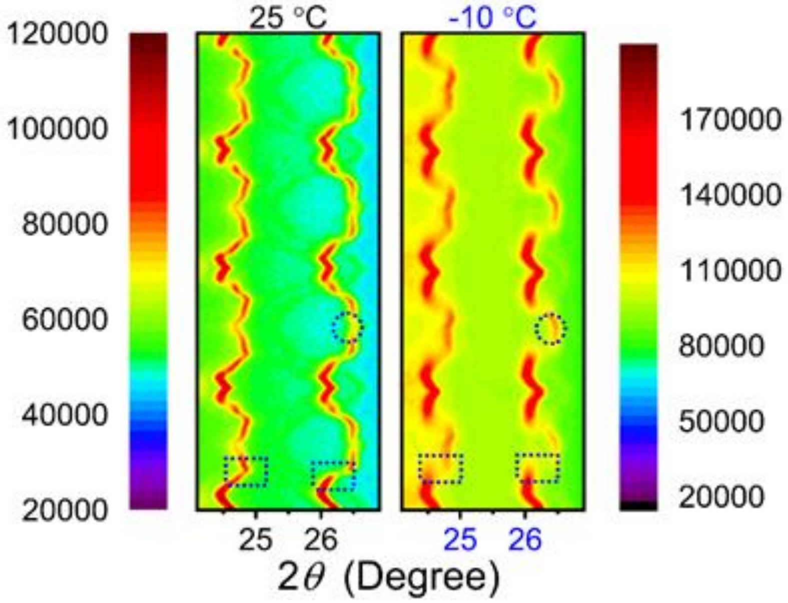

Figure S17. Comparisons of enlarged contour *in-situ* XRD patterns within 24.1–26.9° at 25 and −10 °C.

Table S1. Details of Rietveld refinement and crystal data of $NaNb_{13}O_{33}$.

Sample	Micron-sized particles
Diffractometer	Rigaku Ultima IV
Radiation type	Cu-Kα
Wavelength (Å)	1.5418
Refined profile range (°2θ)	10–70
Step size (°2θ)	0.02

χ^2	6.61
R_p	9.36%
R_{wp}	12.0%
Formula	$NaNb_{13}O_{33}$
Symmetry	Monoclinic
Space group	*C2/m*
a (Å)	22.49614(68)
b (Å)	3.84842(10)
c (Å)	15.42272(42)
β (°)	91.427(2)
Volume (Å^3)	1334.800(83)
Z	2

Table S2. Fractional atomic parameters of $NaNb_{13}O_{33}$ with *C2/m*.

atom	site	*x*	*y*	*z*	occupancy
Na	2*d*	0	0.5	0.5	1
Nb1	2*a*	0	0	0	1
Nb2	4*i*	0.069901	0	0.232653	1
Nb3	4*i*	0.225620	0	0.137090	1
Nb4	4*i*	0.155214	0	0.900358	1
Nb5	4*i*	0.137473	0	0.467459	1
Nb6	4*i*	0.295820	0	0.376773	1
Nb7	4*i*	0.091876	0	0.667963	1
O1	4*i*	0.085069	0	0.945112	1
O2	2*b*	0	0.5	0	1
O3	4*i*	0.368124	0	0.210267	1
O4	4*i*	0.267110	0	0.858763	1
O5	4*i*	0.166229	0	0.182689	1
O6	4*i*	0.473351	0	0.762666	1
O7	4*i*	0.195005	0	0.026912	1
O8	4*i*	0.033984	0	0.126731	1
O9	4*i*	0.134959	0	0.774210	1
O10	4*i*	0.286093	0	0.276447	1
O11	4*i*	0.093866	0	0.365038	1
O12	4*i*	0.010527	0	0.699639	1
O13	4*i*	0.406445	0	0.339860	1
O14	4*i*	0.183126	0	0.614181	1
O15	4*i*	0.228103	0	0.436477	1
O16	4*i*	0.340134	0	0.524395	1
O17	4*i*	0.046246	0	0.544836	1

Table S3. Comparisons of lattice constants of $NaNb_{13}O_{33}$ with that of previously-reported shear ReO_3-type niobates. Interlayer spacings (usually *b* values) are highlighted in red.

Sample	*a* (Å)	*b* (Å)	*c* (Å)	*α*,*γ* (°)	*β* (°)	*V* (Å^3)	reference
**$NaNb_{13}O_{33}$**	**22.49614 (68)**	**3.84842 (10)**	**15.42272 (42)**	**90**	**91.427 (2)**	**1334.800 (83)**	**this work**
$Ni_2Nb_{34}O_{87}$	28.69691 (89)	3.84015 (10)	20.66244 (65)	90	90	2277.011 (167)	S10
$Ga_{0.5}Nb_{24.5}O_{62}$	30.02809 (53)	3.83409 (52)	21.10826 (35)	90	96.041 (1)	2416.709 (927)	S11
$Mg_2Nb_{34}O_{87}$	15.60459 (13)	3.83071 (2)	20.64403 (13)	90	113.096 (6)	1135.119 (161)	S12
$Cr_{0.5}Nb_{24.5}O_{62}$	29.91514 (299)	3.82628 (32)	21.15166 (201)	90	94.944 (8)	2412.092 (488)	S13
$FeNb_{11}O_{29}$	28.70490 (49)	3.82569 (7)	20.62376 (42)	90	90	2264.822 (60)	S14
$W_5Nb_{16}O_{55}$	29.70832 (41)	3.81905 (4)	23.14088 (39)	90	126.546 (6)	2109.270 (547)	S15
$Al_{0.5}Nb_{24.5}O_{62}$	29.9005 (72)	3.8228 (7)	21.1950 (45)	90	95.079 (3)	2413.20 (92)	S16
$ZrNb_{24}O_{62}$	29.87123 (255)	3.82209 (26)	21.16379 0(157)	90	95.078 (7)	2406.798 (430)	S17
$MoNb_{12}O_{33}$	22.7931 (6)	3.82094 (1)	17.72972 (5)	90	123.3 (1)	1261.021 (60)	S18
$Mo_4Nb_{26}O_{77}$	29.76355 (87)	3.82011 (10)	26.00112 (27)	90	92.377 (4)	2953.788 (86)	S19
$Fe_{0.5}Nb_{24.5}O_{62}$	29.7203 (61)	3.81813 (70)	21.1036 (44)	90	95.352 (17)	2383.807 (22)	S20
$Ti_2Nb_{10}O_{29}$	15.5164 (11)	3.8112 (2)	20.5382 (13)	90	113.042 (5)	1117.67 (15)	S21
$TiNb_2O_7$	20.36708 (85)	3.79885 (15)	11.89108 (55)	90	127.227 (3)	794.945 (68)	S22

Table S4. Comparisons of cyclic stability of $NaNb_{13}O_{33}$ with that of previously-reported shear ReO_3-type niobates having similar micron particle sizes.

material	capacity	capacity retention	reference
**$NaNb_{13}O_{33}$**	**120.1 mAh g^{-1} at 2500 mA g^{-1}**	**87.9% after 5000 cycles at 2500 mA g^{-1}**	**this work**
$TiNb_2O_7$	90 mAh g^{-1} at 10C	59.8% after 1000 cycles at 10C	S22
$Ti_2Nb_{10}O_{29}$	80 mAh g^{-1} at 10C	75.0% after 500 cycles at 10C	S21
$TiNb_{24}O_{62}$	147 mAh g^{-1} at 10C	80.9% after 500 cycles at 10C	S23
$MoNb_{12}O_{33}$	138 mAh g^{-1} at 10C	89.2% after 1000 cycles at 10C	S18
$FeNb_{11}O_{29}$	57 mAh g^{-1} at 10C	41.6% after 500 cycles at 10C	S24
$GaNb_{11}O_{29}$	121 mAh g^{-1} at 10C	66.9% after 1000 cycles at 10C	S25
$AlNb_{11}O_{29}$	131 mAh g^{-1} at 10C	93.2% after 500 cycles at 10C	S26
$HfNb_{24}O_{62}$	105 mAh g^{-1} at 10C	87.1% after 500 cycles at 10C	S27
$Mg_2Nb_{34}O_{87}$	149 mAh g^{-1} at 10C	93.5% after 500 cycles at 10C	S12
$Cu_2Nb_{34}O_{87}$	184 mAh g^{-1} at 10C	88.5% after 1000 cycles at 10C	S28
$CrNb_{11}O_{29}$	150 mAh g^{-1} at 10C	90.2% after 400 cycles at 10C	S29
$Mo_3Nb_{14}O_{44}$	123 mAh g^{-1} at 10C	71.8% after 1000 cycles at 10C	S30

Table S5. Comparisons of apparent Li^+ diffusion coefficient (D_{Li}) of $NaNb_{13}O_{33}$ with that of previously-reported shear ReO_3-type niobates.

material	D_{Li} ($cm^2\ s^{-1}$)	test technique	reference
**$NaNb_{13}O_{33}$ micron-sized particles (25 °C)**	**5.86×10^{-11}**	**GITT**	**this work**
**$NaNb_{13}O_{33}$ micron-sized particles (−10 °C)**	**1.94×10^{-11}**	**GITT**	**this work**
**$NaNb_{13}O_{33}$ micron-sized particles (25 °C)**	**2.71×10^{-11}**	**CV**	**this work**
**$NaNb_{13}O_{33}$ micron-sized particles (−10 °C)**	**1.61×10^{-11}**	**CV**	**this work**
$Mg_2Nb_{34}O_{87}$ micron-sized particles (25 °C)	4.80×10^{-13}	CV	S12
$MoNb_{12}O_{33}$ micron-sized particles (25 °C)	3.9×10^{-14}	GITT	S18
$Al_{0.5}Nb_{24.5}O_{62}$ micron-sized particles (25 °C)	2.5×10^{-13}	GITT	S16
$W_5Nb_{16}O_{55}$ micron-sized particles (25 °C)	1.0×10^{-13}	GITT	S16
$AlNb_{11}O_{29}$ micron-sized particles (25 °C)	1.76×10^{-13}	CV	S26
$HfNb_{24}O_{62}$ micron-sized particles (25 °C)	1.61×10^{-12}	GITT	S27
$Cu_2Nb_{34}O_{87}$ micron-sized particles (25 °C)	3.5×10^{-13}	GITT	S28
$TiNb_6O_{17}$ micron-sized particles (25 °C)	4.88×10^{-14}	CV	S31
$Ti_2Nb_{10}O_{27.1}$ micron-sized particles (25 °C)	2.11×10^{-14}	CV	S32
$TiCr_{0.5}Nb_{10.5}O_{29}$ nano-sized particles (25 °C)	2.07×10^{-14}	CV	S33

Table S6. Comparisons of maximum unit-cell-volume variation of $NaNb_{13}O_{33}$ with that of previously-reported shear ReO_3-type niobates.

material	maximum unit-cell-volume change	reference
**$NaNb_{13}O_{33}$**	**6.02% (discharged to 0.8 V)**	**this work**
$Ni_2Nb_{34}O_{87}$	6.71% (discharged to 0.8 V)	S10
$Ti_2Nb_{10}O_{29}$	6.8% (discharged to 1.0 V)	S34
$TiNb_2O_7$	7.22% (discharged to 1.0 V)	S35
$MoNb_{12}O_{33}$	7.8% (discharged to 0.8 V)	S18
$Cu_2Nb_{34}O_{87}$	8.32% (discharged to 0.8 V)	S28
$Al_{0.5}Nb_{24.5}O_{62}$	8.5% (discharged to 0.8 V)	S16
$Zn_2Nb_{34}O_{87}$	8.58% (discharged to 0.8 V)	S15
VNb_9O_{25}	8.91% (discharged to 1.0 V)	S36
$Mo_3Nb_{14}O_{44}$	10.6% (discharged to 0.8 V)	S30
PNb_9O_{25}	10.69% (discharged to 1.0 V)	S37
$Mo_4Nb_{26}O_{77}$	12.8% (discharged to 0.8 V)	S19
$TiNb_{24}O_{62}$	17.5% (discharged to 1.0 V)	S38

References

[S1] B. Toby, EXPGUI, a graphical user interface for GSAS, J. Appl. Crystallogr. 34 (2001) 210–213.

[S2] W. Wang, Q. Zhang, T. Jiang, S. Li, J. Gao, X. Liu, C. Lin, Conductive $LaCeNb_6O_{18}$ with a very open A-site-cation-deficient perovskite structure: a fast- and stable-charging Li^+-storage anode compound in a wide temperature range, Adv. Energy Mater. 12 (2022) 2200656.

[S3] G. Liang, L. Yang, X. Xiong, K. Pei, X. Zhao, C. Wang, W. You, X. Liu, X. Zhang, R. Che, Interfacial space charge enhanced sodium storage in a zero-Strain cerium niobite perovskite anode, Adv. Funct. Mater. 32 (2022) 2206129.

[S4] J. Hafner, Ab-initio simulations of materials using VASP: density-functional theory and beyond, J. Comput. Chem. 29 (2008) 2044–2078.

[S5] G. Kresse, J. Furthmuller, Efficient iterative schemes for ab initio total-energy calculations using a plane-wave basis set, Phys. Rev. B 54 (1996) 11169–11186.

[S6] J.P. Perdew, K. Burke, M. Ernzerhof, Generalized gradient approximation made simple, Phys. Rev. Lett. 77 (1996) 3865–3868.

[S7] G. Henkelman, B. Uberuaga, P. Jonsson, Hannes, A climbing image nudged elastic band method for finding saddle points and minimum energy paths, J. Chem. Phys. 113 (2000) 9901–9904.

[S8] V.G. Zubkov, L.L. Surat, A.P. Tyutyunnik, I.F. Berger, M.Y. Skripkin, Structural, vibrational, electronic, and luminescence properties of the cyclotetravanadates $A_2M(VO_3)_4$ (A=Na, Ag; M=Ca, Sr), Phys. Rev. B 77 (2008) 174113.

[S9] A.J. Bard, L.R. Faulkner, Electrochemical Methods: Fundamentals and Applications, 2nd edn., Wiley, New York, USA 2001.

[S10] C. Lv, C. Lin, X. Zhao, Rational design and synthesis of nickel niobium oxide with high-rate capability and cycling stability in a wide temperature range, Adv. Energy Mater. 12 (2022) 2102550.

[S11] R. Li, Y. Pu, J. Xu, Q. Fu, G. Liang, X. Zhu, L. Luo, Y. Chen, C. Lin, Novel $GaNb_{49}O_{124}$ microspheres with intercalation pseudocapacitance for ultrastable lithium-ion storage, Ceram. Int. 45 (2019) 12211–12217.

[S12] X. Zhu, Q. Fu, L. Tang, C. Lin, J. Xu, G. Liang, R. Li, L. Luo, Y. Chen, $Mg_2Nb_{34}O_{87}$ porous microspheres for use in high-energy, safe, fast-charging, and stable lithium-ion batteries, ACS Appl. Mater. Interfaces 10 (2018) 23711–23720.

[S13] C. Yang, S. Yu, C. Lin, F. Lv, S. Wu, Y. Yang, W. Wang, Z. Zhu, J. Li, N. Wang, S. Guo, $Cr_{0.5}Nb_{24.5}O_{62}$ nanowires with high electronic conductivity for high-rate and long-life lithium-ion storage, ACS Nano 11 (2017) 4217–4224.

[S14] X. Lou, C. Lin, Q. Luo, J. Zhao, B. Wang, J. Li, Q. Shao, X. Guo, N. Wang, Z. Guo, Crystal structure modification enhanced $FeNb_{11}O_{29}$ anodes for lithium-ion batteries, ChemElectroChem 4 (2017) 3171–3180.

[S15] X. Zhu, H. Cao, R. Li, Q. Fu, G. Liang, Y. Chen, L. Luo, C. Lin, X. Zhao, Zinc niobate materials: crystal structures, energy-storage capabilities and working mechanisms, J. Mater. Chem. A 7 (2019) 25537–25547.

[S16] Q. Fu, R. Li, X. Zhu, G. Liang, L. Luo, Y. Chen, C. Lin, X. Zhao, Design, synthesis and lithium-ion storage capability of $Al_{0.5}Nb_{24.5}O_{62}$, J. Mater. Chem. A 7 (2019) 19862–19871.

[S17] C. Yang, Y. Zhang, F. Lv, C. Lin, Y. Liu, K. Wang, J. Feng, X. Wang, Y. Chen, J. Li, S. Guo, Porous $ZrNb_{24}O_{62}$ nanowires with pseudocapacitive behavior achieve high-performance lithium-ion storage, J. Mater. Chem. A 5 (2017) 22297–22304.

[S18] X. Zhu, J. Xu, Y. Luo, Q. Fu, G. Liang, L. Luo, Y. Chen, C. Lin, X. Zhao, $MoNb_{12}O_{33}$ as a new anode material for high-capacity, safe, rapid and durable Li^{+} storage: structural characteristics, electrochemical properties and working mechanisms, J. Mater. Chem. A 7 (2019) 6522–6532.

[S19] S. Li, J. Gao, Y. Ou, W. Wang, Q. Zhang, S. Gao, X, Liu, C. Lin, A general strategy to enhance the electrochemical activity and energy density of energy-storage materials through using sintering aids with redox activity: a case study of $Mo_4Nb_{26}O_{77}$, J. Mater. Chem. A. 10 (2022) 19953–19962.

[S20] R. Zheng, Y. Li, H. Yu, T. Liu, M. Xia, X. Zhang, N. Peng, J. Zhang, Y. Bai, J. Shu, Rational construction and decoration of $Fe_{0.5}Nb_{24.5}O_{62-x}$@C nanowires as superior anode material for lithium storage, Chem. Eng. J. 384 (2020) 123314.

[S21] C. Yang, S. Yu, Y. Ma, C. Lin, Z. Xu, H. Zhao, S. Wu, P. Zheng, Z.Z. Zhu, J. Li, N. Wang, Cr^{3+} and Nb^{5+} co-doped $Ti_2Nb_{10}O_{29}$ materials for high-performance lithium-ion storage, J. Power Sources 360 (2017) 470–479.

[S22] C. Yang, C. Lin, S. Lin, Y. Chen, J. Li, $Cu_{0.02}Ti_{0.94}Nb_{2.04}O_7$: an advanced anode material for lithium-ion batteries of electric vehicles, J. Power Sources 328 (2016) 336–344.

[S23] C. Yang, S. Deng, C. Lin, S. Lin, Y. Chen, J. Li, H. Wu, Porous $TiNb_{24}O_{62}$ microspheres as high-performance anode materials for lithium-ion batteries of electric vehicles, Nanoscale 8 (2016) 18792–18799.

[S24] X. Lou, Z. Xu, Z. Luo, C. Lin, C. Yang, H. Zhao, P. Zheng, J. Li, N. Wang, Y. Chen, H. Wu, Exploration of $Cr_{0.2}Fe_{0.8}Nb_{11}O_{29}$ as an advanced anode material for lithium-ion batteries of electric vehicles, Electrochim. Acta 245 (2017) 474–480.

[S25] X. Lou, Q. Fu, J. Xu, X. Liu, C. Lin, J. Han, Y. Luo, Y. Chen, X. Fan, J. Li, $GaNb_{11}O_{29}$ nanowebs as high-performance anode materials for lithium-ion batteries, ACS Appl. Nano Mater. 1 (2018) 183–190.

[S26] X. Lou, R. Li, X. Zhu, L. Luo, Y. Chen, C. Lin, H. Li, X. Zhao, New anode material for lithium-ion batteries: aluminum niobate ($AlNb_{11}O_{29}$), ACS Appl. Mater. Interfaces 11 (2019) 6089–6096.

[S27] Q. Fu, H. Cao, G. Liang, L. Luo, Y. Chen, V. Murugadoss, S. Wu, T. Ding, C. Lin, Z. Guo, A highly Li^+-conductive $HfNb_{24}O_{62}$ anode material for superior Li^+ storage, Chem. Commun. 56 (2020) 619–622.

[S28] L. Yang, X. Zhu, X. Li, X. Zhao, K. Pei, W. You, X. Li, Y. Chen, C. Lin, R. Che, Conductive copper niobate: superior Li^+-storage capability and novel Li^+-transport mechanism, Adv. Energy Mater. 9 (2019) 1920174.

[S29] Q. Fu, X. Liu, J. Hou, Y. Pu, C. Lin, L. Yang, X. Zhu, L. Hu, S. Lin, L. Luo, Y. Chen, Highly conductive $CrNb_{11}O_{29}$ nanorods for use in high-energy, safe, fast-charging and stable lithium-ion batteries, J. Power Sources 397 (2018) 231–239.

[S30] R. Li, G. Liang, X. Zhu, Q. Fu, Y. Chen, L. Luo, C. Lin, $Mo_3Nb_{14}O_{44}$: a new Li^+ container for high-performance electrochemical energy storage, Energy Environ. Mater. 4 (2021) 65–71.

[S31] C. Lin, G. Wang, S. Lin, J. Li, L. Lu, $TiNb_6O_{17}$: a new electrode material for lithium-ion batteries, Chem. Commun. 51 (2015) 8970–8973.

[S32] C. Lin, S. Yu, H. Zhao, S. Wu, G. Wang, L. Yu, Y. Li, Z. Zhu, J. Li, S. Lin, Defective $Ti_2Nb_{10}O_{27.1}$: an advanced anode material for lithium-ion batteries, Sci. Rep. 5 (2015) 17836.

[S33] L. Hu, R. Lu, L. Tang, R. Xia, C. Lin, Z. Luo, Y. Chen, J. Li, $TiCr_{0.5}Nb_{10.5}O_{29}$/CNTs nanocomposite as an advanced anode material for high-performance Li^+-ion storage, J. Alloys Compd. 732 (2018) 116–123.

[S34] H. Li, X. Cai, J. Li, C. Deng, Y. Liu, H. Yan, H. Yu, L. Zhang, M. Shui, L. Yan, J. Shu, $Ti_2Nb_{10}O_{29}$@C hollow submicron ribbons for superior lithium storage, Ceram. Int. 48 (2022) 23334–23340.

[S35] B. Guo, X. Yu, X. Sun, M. Chi, Z. Qiao, J. Liu, Y. Hu, X. Yang, J.B. Goodenough, S. Dai, A long-life lithium-ion battery with a highly porous $TiNb_2O_7$ anode for large-scale electrical energy storage, Energy Environ. Sci. 7 (2014) 2220–2226.

[S36] S. Qian, H. Yu, L. Yan, H. Zhu, X. Cheng, Y. Xie, N. Long, M. Shui, J. Shu, High-rate long-life pored nanoribbon VNb_9O_{25} built by interconnected ultrafine nanoparticles as anode for lithium-ion batteries, ACS Appl. Mater. Interfaces 9 (2017) 30608–30616.

[S37] H. Yu, J. Zhang, R. Zheng, T. Liu, N. Peng, Y. Yuan, Y. Liu, J. Shu, Z. Wang, The journey of lithium ions in the lattice of PNb_9O_{25}, Mater. Chem. Front. 4 (2020) 631–637.

[S38] H. Yu, X. Cheng, H. Zhu, R. Zheng, T. Liu, J. Zhang, M. Shui, Y. Xie, J. Shu, Deep insights into kinetics and structural evolution of nitrogen-doped carbon coated $TiNb_{24}O_{62}$ nanowires as high-performance lithium container, Nano Energy 54 (2018) 227–237.
